# Effects of Docetaxel Injection and Docetaxel Micelles on the Intestinal Barrier and Intestinal Microbiota

**DOI:** 10.1002/advs.202102952

**Published:** 2021-10-28

**Authors:** Qingya Liu, Yi Lu, Yao Xiao, Liping Yuan, Danrong Hu, Ying Hao, Ruxia Han, Jinrong Peng, Zhiyong Qian

**Affiliations:** ^1^ State Key Laboratory of Biotherapy and Cancer Center West China Hospital Sichuan University and Collaborative Innovation Center of Biotherapy Chengdu Sichuan 610041 P. R. China; ^2^ West China School of Pharmacy Sichuan University Chengdu 610041 P. R. China

**Keywords:** chemotherapy, docetaxel, drug formulation, intestinal barrier, intestinal microbiota

## Abstract

Increasing evidence has suggested that chemotherapeutics affect the integrity of the intestinal barrier and alter the intestinal microbiota, thus limiting the therapeutic outcomes of cancer chemotherapy. Docetaxel (DTX) is used for breast cancer treatment and has gastrointestinal side effects, but the influence of DTX formulations on the intestinal barrier and intestinal microbiota remains unknown. Therefore, in this work, the influence of DTX injection (free DTX, commercial formulation) and DTX/methoxy poly(ethylene glycol)‐*b* *lock*‐poly(D,L‐lactide) (mPEG‐PDLLA) (DTX micelles, nanoformulation) on the integrity of the intestinal barrier and the intestinal microbiota is investigated. It is found that the free DTX causes significantly greater intestinal barrier damage than the DTX micelles. The diversity of the intestinal microbiota, and the relative abundance of *Akkermansia muciniphila* and *Ruminococcus gnavus* in the DTX micelle‐treated group is significantly higher than that in the free DTX‐treated group. Moreover, the tumor growth rate is elevated in antibiotic mixture‐pretreated mice, demonstrating that the diversity and composition of the intestinal microbiota may be associated with tumor progression. This work demonstrates that different formulations of chemotherapeutics have different effects on the integrity of the intestinal barrier and the intestinal microbiota.

## Graphical abstract

The effects of chemotherapeutic formulations onto the intestinal barrier integrity and intestinal microbiota are evaluated. It reveals that the docetaxel (DTX)/mPEG‐PDLLA micelles cause lower damage to the intestinal barrier integrity than free form DTX, and the micelles are also conducive to the intestinal microbiota diversity and probiotics enrichment than free form DTX. It favors the development of more suitable nanomedicines.

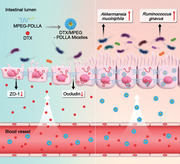

## 1 Introduction

DTX is a chemotherapy drug that is often used for breast cancer treatment.^[^ ^1^ ^]^ It can effectively inhibit the proliferation of rapidly growing cells by stabilizing the microtubule during mitosis.^[^ ^2^ ^]^ However, DTX itself and its adjuvant polysorbate 80 can cause many side effects.^[^ ^3^, ^4^ ^]^ The main side effects include “off‐target” toxicity, myelosuppression, vomiting, diarrhea, stomatitis, and colitis.^[^ ^5^ ^]^ In general, the gastrointestinal mucosa is easily inhibited by chemotherapeutic drugs due to its high cellular turnover, which leads to chemotherapy‐associated gastrointestinal side effects.^[^ ^6^ ^]^ Notably, nanotechnology has begun to be used to construct nanomedicines, for example, liposomes, nanogel, and micelles, which are expected to alleviate the adverse effects of chemotherapeutics.^[^ ^7^, ^8^, ^9^, ^10^ ^]^ Indeed, some side effects, especially the cardiotoxicity and allergic reactions, have already been mitigated.^[^ ^11^, ^12^ ^]^ In the case of DTX, the side effects of DTX micelles are less severe than those of free DTX, and these micelles have been shown to exhibit better antitumor efficacy than free DTX in breast cancer therapy.^[^ ^3^, ^13^, ^14^, ^15^ ^]^ Nonetheless, most previous studies have mainly focused on evaluating the potential of nanocarriers to reduce “off‐targeted” toxicity; the influences of the formulations on the gastrointestinal environment have not been thoroughly investigated.

The intestinal barrier and intestinal microbiota are two important components of the intestinal environment. The intestinal barrier consists of an epithelial layer in which the cells are recycled every 3–5 days to replace older and compromised cells. Epithelial cells are bound by tight junctions to form and maintain the integrity of the intestinal barrier, and they form the physical barrier of the intestine.^[^ ^16^ ^]^ Tight junctions are composed of a variety of proteins, including occludin and zonula occludens‐1 (ZO‐1).^[^ ^17^ ^]^ By forming a physical barrier, a complete intestinal epithelium not only helps to maintain the stability of the intestinal environment but also facilitates nutrient absorption and waste secretion.^[^ ^18^ ^]^ However, intestinal damage may lead to local and systemic diseases, including celiac disease and inflammatory bowel disease.^[^ ^19^, ^20^ ^]^ Although the important role of the intestinal barrier in normal intestinal physiology has been thoroughly demonstrated, and it is reported that DTX may cause intestinal barrier damage, which increase intestinal permeability, and the changes in intestinal permeability are associated with the gastrointestinal toxicity;^[^ ^21^, ^22^, ^23^, ^24^ ^]^ however, few studies have evaluated the effects of the DTX formulations on the permeability or integrity of the intestinal barrier.

In addition, intestinal microbiota is another main component of the gastrointestinal environment. Some studies have reported that chemotherapeutic drugs likely change the intestinal microbiota, which in turn may affect the efficacy and gastrointestinal toxicity of chemotherapy.^[^ ^25^ ^]^ The intestinal microbiota is an ecosystem with high complexity and diversity. With increasing understanding of this population, its important roles in normal physiological processes and disease progression have been gradually recognized.^[^ ^26^, ^27^ ^]^ Studies have shown that the intestinal microbiota plays a crucial role in the absorption of nutrients, the regulation of intestinal intrinsic neural networks, the efficacy and side effects of chemotherapeutics, immunity, and other processes.^[^ ^28^ ^]^ In particular, recent work on preclinical models has emphasized the importance of the intestinal microbiota in modifying tumor responses to chemotherapeutic drugs.^[^ ^29^ ^]^ The diversity and abundance of intestinal microbes are the main indicators used to evaluate fluctuations in the intestinal microbiota after treatments, as changes in the diversity of microbiota may be connected with therapeutic outcomes. For example, Winer et al. found that high‐fat diet‐induced diabetes with reduced IgA immune cell numbers and IgA secretion leads to altered diversity of the intestinal microbiota.^[^ ^30^ ^]^ Beyond diversity of the microbiota as a whole, the abundance of specific intestinal bacteria may also be affected as the disease develops. Based on the influences of some strains on disease and treatment outcomes, probiotics have been used in adjunctive therapies for chemotherapy in the treatment of cancer.^[^ ^31^ ^]^ Probiotics can protect the intestinal epithelium from radiation injury and prevent or ameliorate the toxic effects of anticancer therapies.^[^ ^32^, ^33^ ^]^ Moreover, the intestinal microbiota has been implicated in the metabolism of many drugs and resulting in drug toxicity. The activities of chemotherapeutics may change the abundance of specific bacterial species and the diversity of the intestinal microbiota, and vice versa.^[^ ^25^ ^]^ However, there is still a lack of studies on the effects of DTX formulations on the intestinal microbiota and its diversity at the genus and species levels.

**Scheme 1:**
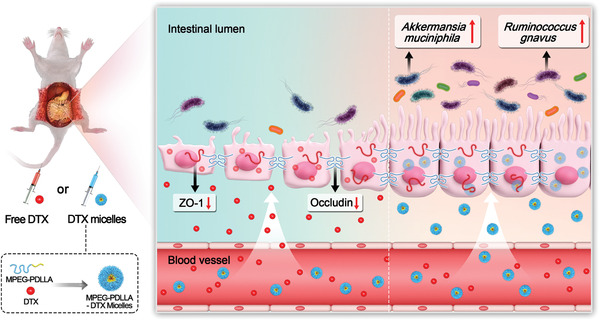
Impact of DTX on the intestinal barrier and the intestinal microbiota. DTX in two dosage forms was administered nonorally. The drug entered the intestine from the blood vessels and affected the integrity of the intestinal barrier and the composition of the intestinalmicrobiota.

Therefore, in view of the gastrointestinal toxicity of free DTX, the superior antitumor efficacy of DTX micelles, the role of the intestinal microbiota in cancer therapy, and the improvements in side effects offered by probiotics, investigations into the changes in the intestinal microbiota that occur free DTX and DTX micelle treatment are needed. Importantly, the intestinal microbiota changes with the development of some diseases. Cancer is caused by complex interactions between the host and the environment.^[^ ^34^ ^]^ Thus far, related research has mainly focused on the effects of the intestinal microbiota on cancer; few studies have investigated whether cancer leads to changes in the intestinal microbiota. Herein, DTX micelles were prepared and characterized according to our previous report.^[^ ^3^, ^35^ ^]^ Then, we compared the influences of DTX micelles and free DTX on the integrity of the intestinal barrier in vitro and in vivo. Furthermore, we investigated the influences of free DTX and DTX micelles on intestinal microbial diversity and the intestinal microbiota composition at the genus and species levels (**Scheme** **1**). Finally, we evaluated the changes in the intestinal microbiota that occur during cancer development and the effect of microbiota manipulation with antibiotics on the progression of cancer, as antibiotics can cause large and highly variable changes in the intestinal microbiota.^[^ ^36^ ^]^ We hope this study can provide useful information about the effects of drug formulations on the gastrointestinal environment, thus providing some guidance for the evaluation and construction of nanomedicines.

## 2 Results and Discussion

### 2.1 Characterization of the DTX Micelles

**Figure 1:**
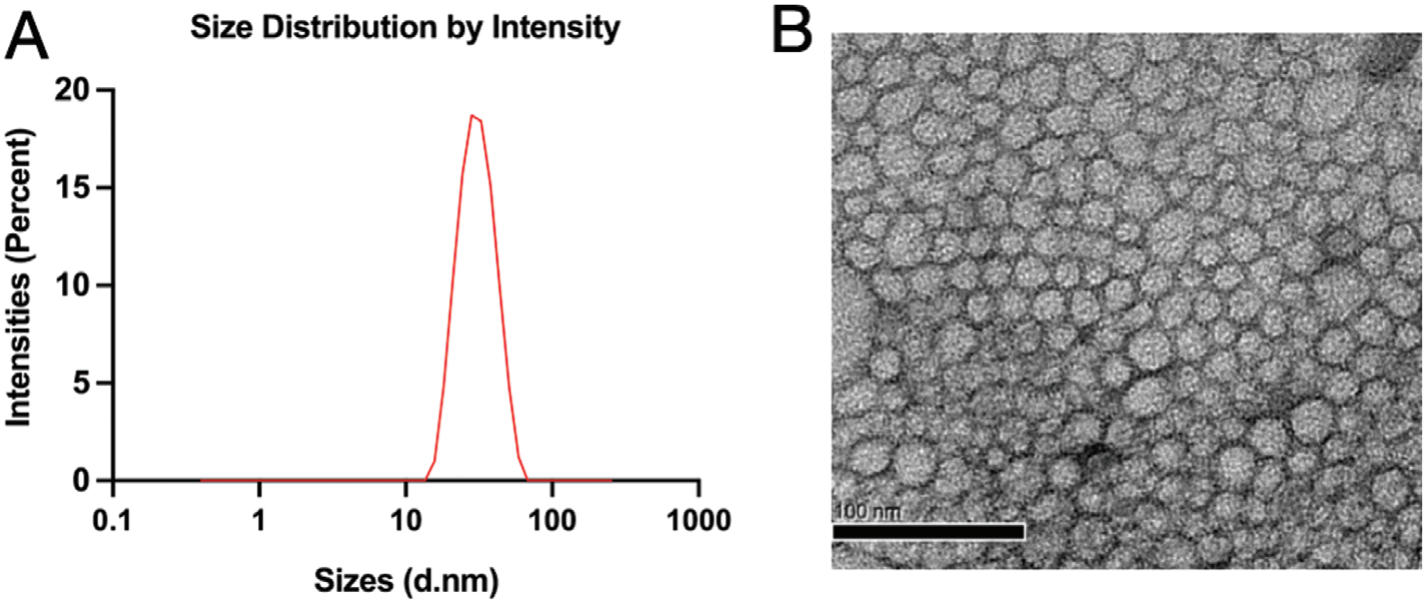
Characterization of DTX micelles. A) Particle distributions of DTX micelles measured with a Malvern Zetasizer Nano ZS. B) Morphology of DTXunder transmission electron microscopy (TEM) (scale bar = 100 nm).

DTX is a common chemotherapy drug for breast cancer, and DTX micelles has better efficacy than free DTX with fewer side effects.^[^ ^3^ ^]^ In this study, the average diameter of the DTXmicelles was 29.07±0.28 nm, and the polydispersity index was 0.086±0.019, with a highly uniform and narrow distribution (**Figure** **1**A). The morphology of the DTX micelles is shown in Figure 1B; the diameter of the micelles was similar to that obtained with the Zetasizer data. These findings were consistent with those of our previous studies.^[^ ^3^ ^]^

### 2.2 In Vivo Biodistribution and Intestinal Barrier Permeability

**Figure 2:**
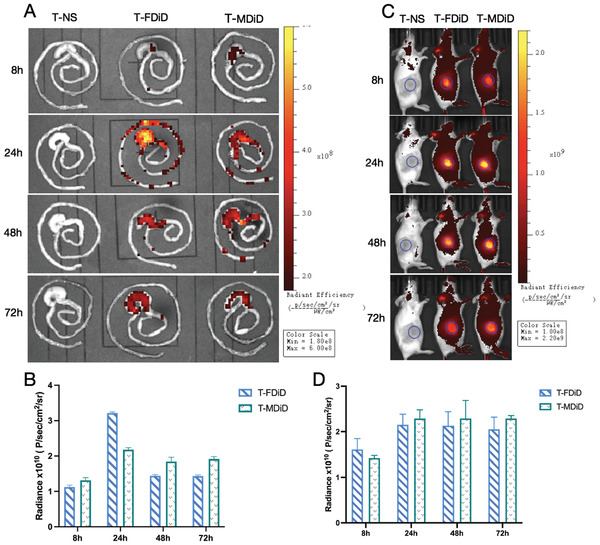
Distributions of free DTX and DTX micelles in vivo. T‐NS, T‐FDiD, and T‐MDiD represent tumor‐bearing mice treated with normal saline, free DiD, and DiD micelles, respectively. A) Distributions of free DiD and DiD micelles in the intestine 8 to 72 h after injection. B) Quantitative analysis of intestinal fluorescence intensity at set time points after injection (three mice were detected at each time point, mean ± SD, Student's *t*‐test). C) Distributions of free DiD and DiD micelles in tumors 8 to 72 h after injection. D) Quantitative analysis of tumor fluorescence intensity at set time points (three mice were detected at each time point, mean ± SD, Student's *t*‐test).

Although DTX was administered intravenously to mice, we studied whether DTX was distributed in the intestine. DiD was used instead of DTX as the fluorescent chromogenic agent in the micelles, and the distribution of free DiD and DiD micelles in the intestine was detected with a fluorescence imaging system. Within 8 and 72 h after injection of the same dose of DiD, DiD fluorescence was observed in intestine and tumor tissues. The fluorescence intensity in the mouse intestine was the strongest at 24 h, and the fluorescence intensity of free DiD was stronger than that of DiD micelles (**Figure** **2**A,B); however, at the tumor site, the fluorescence intensity of free DiD at the 24‐h time point was lower than that of DiD micelles (Figure 2C,D). The fluorescence intensity of free DiD in the tumor tissue did not exceed that of DiD micelles at 24 h or afterward. These findings demonstrate the advantage of nanoparticles for targeted drug delivery.^[^ ^37^ ^]^

**Figure 3:**
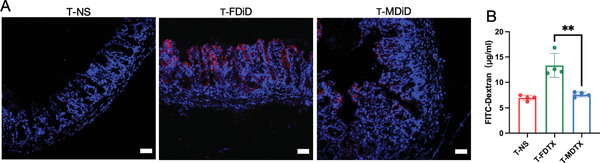
Distributions of free DTX and DTX micelles in the colon and the intestinal permeability of the colon. A) Images of frozen sections showing the distribution of DiD in the villi 24 h after injection (scale bar = 50 µm). Blue fluorescence represents the nucleus, and red fluorescence represents DiD. B) Intestinal barrier permeability (*n* = 4, mean ± SD, Student's *t*‐test, *p* = 0.0032, ^**^ *p* < 0.01). T‐NS, T‐FDTX, and T‐MDTX represent tumor‐bearing mice treated with normal saline, free DTX, and DTX micelles, respectively.

Next, we evaluated the distribution of DiD in the colon at 24 h using frozen sections. The results showed that DiD was mainly distributed in intestinal villi, and the fluorescence intensity of free DiD was stronger than that of DiD micelles (**Figure** **3**A), which was consistent with the distribution of DiD in the whole intestine (Figure 2A,B). The intestinal barrier is the first line of host defense against invading enteric pathogenic bacteria and toxins.^[^ ^38^ ^]^ Some drugs can affect the integrity of the intestinal barrier; subsequently, passage of enteric pathogenic bacteria and toxins through the intestinal barrier can result in damage to other tissues. Thus, damage to the intestinal barrier results in increased intestinal permeability.^[^ ^39^ ^]^ Mouse intestinal permeability was higher in the free DTX group than in the DTX micelle group (Figure 3B), which indicated that free DTX caused greater damage to the intestinal barrier than the DTX micelles.

### 2.3 In Vivo and In Vitro Barrier Assessment

**Figure 4:**
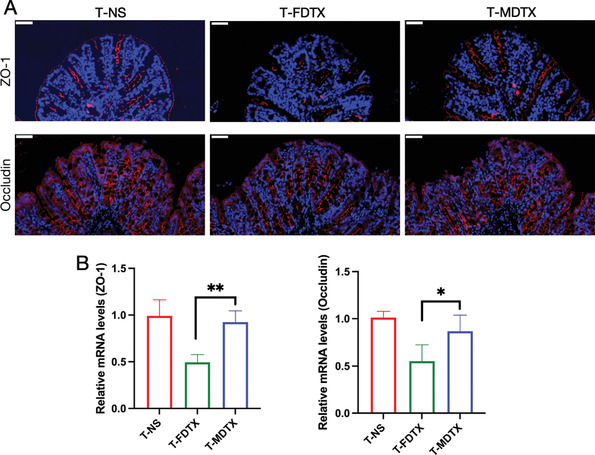
Free DTX and DTX micelles impact the intestinal barrier in vivo. T‐NS, T‐FDTX, and T‐MDTX represent tumor‐bearing mice treated with normal saline, free DTX, and DTX micelles, respectively. A) Immunofluorescence staining analysis of ZO‐1 (red) and occludin (red) expression in the intestine; blue fluorescence represents the nucleus (scale bar = 50 µm). B) mRNA levels of ZO‐1 (*p* = 0.0071) and occludin (*p* = 0.0416) in the colon (*n* = 4, mean ± SD, Student's *t*‐test, ^*^ *p* < 0.05, ^**^ *p* < 0.01).

The permeability and barrier function of the intestinal epithelium depend on the regulation of intercellular tight junctions.^[^ ^40^ ^]^ Recent studies have shown that ZO‐1 and occludin are important in the leak pathway of the intestine.^[^ ^41^, ^42^ ^]^ After observing that the two formulations of DTX had different effects on intestinal permeability, we examined the tight junction proteins ZO‐1 and occludin in the colon. Immunofluorescence analysis showed that ZO‐1 and occludin protein levels in the free DTX group were lower than those in the DTX micelle group (**Figure** **4**A). In addition, the expression patterns and mRNA levels of ZO‐1 and occludin in the colon (Figure 4B) were consistent with the immunofluorescence analysis results, which confirmed that free DTX caused greater damage to the intestinal barrier than the DTX micelles.

**Figure 5:**
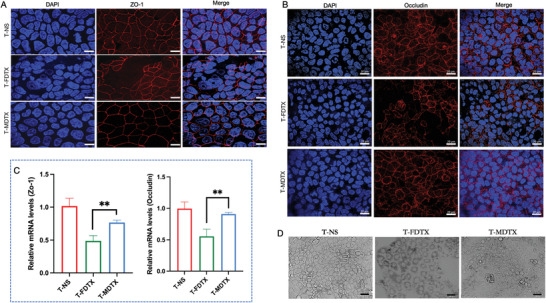
Free DTX and DTX micelles impact the intestinal barrier in vitro. A) Immunofluorescence staining analysis of ZO‐1expression in the intestine (scale bar = 20 µm). B) Immunofluorescence staining analysis of occludin expression in the intestine (scale bar = 20 µm). C) mRNA levels of ZO‐1 (*p* = 0.0061) and occludin (*p* = 0.0056) in Caco‐2 cells (*n* = 4, mean ± SD, Student's *t*‐test, ^**^ *p* < 0.01). D) Impact of free DTX and DTX micelles on the morphology of Caco‐2 cells (scale bar = 50 µm).

To verify the damage to the intestinal barrier caused by the two formulations of DTX, Caco‐2 cells were treated with free DTX and DTX micelles, and the distribution and expression of ZO‐1 and occludin were detected by immunofluorescence analysis and quantitative real‐time PCR (qPCR). Immunofluorescence analysis showed that ZO‐1 and occludin were located around the Caco‐2 cell perimeter. Free DTX treatment resulted in the absence of ZO‐1 and occludin around the cell clusters; however, DTX micelles treatment had little effect on the cells (**Figure** **5**A,B). The mRNA levels of ZO‐1 and occludin in Caco‐2 cells treated with free DTX were significantly lower than those in cells treated with DTX micelles (Figure 5C). Observation of Caco‐2 cell morphology under a microscope revealed that free DTX treatment caused the distance between cells to increase and the boundaries of cells to became blurred, while DTX micelle treatment had little effect on Caco‐2 cell morphology (Figure 5D). These results indicated that free DTX damaged the intestinal barrier to a greater extent than the DTX micelles. Notably, previous studies have observed that the hydrophilic shell of mPEG‐PDLLA maintains micelles in a dispersed state and decreases undesirable drug interactions with cells and proteins.^[^ ^43^, ^44^ ^]^ In addition, DTX micelles have good water solubility, which eliminates the need for Tween 80 to increase the dissolution of DTX and thus reduces damage to the intestinal barrier.^[^ ^45^ ^]^ This may be the reason that the DTX micelles caused less intestinal barrier damage than free DTX.

### 2.4 Modulation of the Intestinal Microbiota

**Figure 6:**
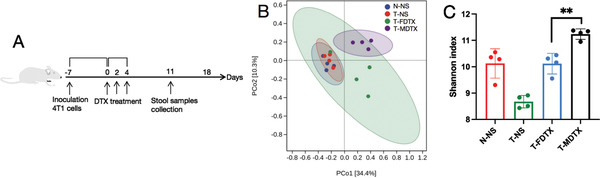
Changes in the intestinal microbiota induced by free DTX or DTX micelles. N‐NS represents normal mice, while T‐NS, T‐FDTX, and T‐MDTX represent tumor‐bearing mice treated with normal saline, free DTX, and DTX micelles, respectively (*n* = 4). A) Outline of the mouse experiment. B) PCA showing differences in the microbial communities between samples. Each point represents a mouse. C) Changes in intestinal microbiota diversity (Shannon index, mean ± SD, Student's *t*‐test, *p* = 0.0022, ^**^ *p* < 0.01).

Previous studies have reported that drugs can alter the intestinal bacterial community directly^[^ ^46^, ^47^ ^]^ and that there are interactions between drugs and individual intestinal bacteria.^[^ ^48^ ^]^ Based on the aggregation of DTX in the intestine, we compared the intestinal microbiota composition after free DTX and DTX micelle treatment at the sampling times shown in **Figure** **6**A. In these experiments, the mice were allowed to acquire microbes from the microenvironment of the cage. To evaluate the impacts of different DTX formulations on the intestinal microbiota, we used Bray–Curtis analysis to perform principal component analysis (PCA) on the basis of operational taxonomic units (OTUs). The microbial communities of DTX micelle‐treated mice differed from those of free DTX‐treated mice (Figure 6B). According to the Shannon index, the intestinal microbiota was more diverse in DTX micelle‐treated mice than in free DTX ‐treated mice (Figure 6C). Recent studies have revealed that the diversity of the intestinal microbiota is associated with the occurrence and treatment of disease, with higher diversity contributing to better health.^[^ ^49^, ^50^ ^]^ In one study, there were significant differences in the diversity and composition of the intestinal microbiota between responders and nonresponders among melanoma patients receiving anti‐PD‐1 therapy, and patients with a high intestinal microbiota diversity had more favorable clinical outcomes after anti‐PD‐1 immunotherapy.^[^ ^51^ ^]^

**Figure 7:**
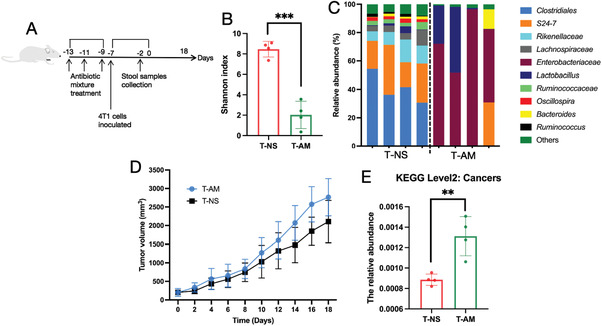
Changes in the intestinal microbiota induced by free DTX or DTX micelles. T‐NS and T‐AM represent tumor‐bearing mice treated with normal saline and an antibiotic mixture, respectively (*n* = 4). A) Outline of the mouse experiment. B) Changes in intestinal microbiota diversity (Shannon index, mean ± SD, Student's *t*‐test, *p* = 0.0002, ^***^ *p* < 0.001). C) Composition and abundance of the intestinal microbiota at the genus level. D) Tumor growth curves. E) Predicted cancers for the metagenome of the intestinal microbiota in each group shown with KEGG level 2 (mean ± SD, Student's *t*‐test, *p* = 0.0053, ^**^ *p* < 0.01).

Based on the difference in the intestinal microbiota diversity between the free DTX‐treated and DTX micelle‐treated groups, we next eliminated the intestinal microbiota with an antibiotic mixture and investigated the effect on tumor growth. We gavaged mice with the antibiotic mixture and then inoculated them with the 4T1 cell line (**Figure** **7**A). We found that the diversity of the intestinal microbiota was significantly decreased after antibiotic mixture treatment (Figure 7B). We also observed that *Enterobacteriaceae* overgrowth after antibiotic mixture treatment (Figure 7C). Notably, the tumor growth rate of the antibiotic mixture group was faster than that of the normal saline group (Figure 7D), and at KEGG level 2, metagenomic prediction of cancer association in the intestinal microbiota in antibiotic mixture‐treated mice was higher than that in normal saline‐treated mice (Figure 7E). Similarly, Bower et al. found that the use of antibiotics before immune checkpoint inhibitor (ICI) treatment was associated with adverse reactions and a worse survival rate among patients receiving ICI treatment.^[^ ^13^ ^]^ In addition, a recent study reported the occurrence of *Enterobacteriaceae* overgrowth with altered tumor development during the progression of inflammation in Il10^−^/^−^ mice.^[^ ^52^ ^]^ These results show that the diversity of the intestinal microbiota and the abundance of some genera may be associated with tumor growth.

**Figure 8:**
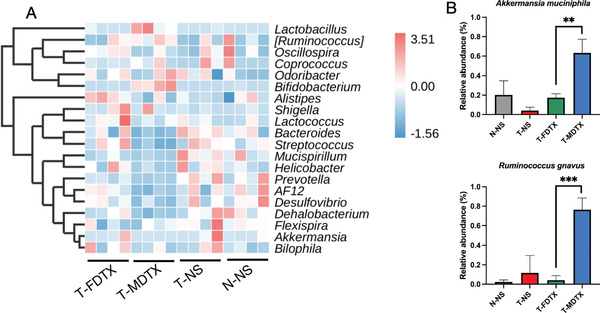
Composition and relative abundance of the intestinal microbiota after treatment with free DTX or DTX micelles. A) Heatmap of the relative abundance of genus‐level taxa (*n* = 4). B) Relative abundance of *Akkermansia muciniphila* (*p* = 0.0057) and *Ruminococcus gnavus* (*p* = 0.0006) (*n* = 4, mean ± SD, Student's *t*‐test, ^**^ *p* < 0.01, ^***^ *p* < 0.001).

The diversity after free DTX treatment was similar to that of normal mice, but the therapeutic outcome was different from that after DTX micelles treatment, and the antibiotic mixture changed the intestinal microbiota structure and then showed the changes of tumor growth rate. Therefore, we further analyzed the composition and abundance of the intestinal microbiota at the genus and species levels. At the genus level, DTX micelles increased the relative abundance of *Bifidobacterium* (**Figure** **8**A), strains of which can improve intestinal barrier integrity.^[^ ^53^ ^]^ In addition, DTX micelle treatment significantly increased the relative abundance of *Akkermansia muciniphila* and *Ruminococcus gnavus* (Figure 8B). *A. muciniphila* is associated with intestinal barrier protection, and it is also beneficial for immunotherapy.^[^ ^54^ ^]^ When lysozyme production in Paneth cells is destroyed, *R. gnavus* supplementation can induce a type 2 immune response, reprogram epithelial cells, and promote tissue healing.^[^ ^55^ ^]^ However, it remains to be further studied whether *A. muciniphila* and *R. gnavus* are beneficial for DTX micelle‐mediated protection of the intestinal barrier.

**Figure 9:**
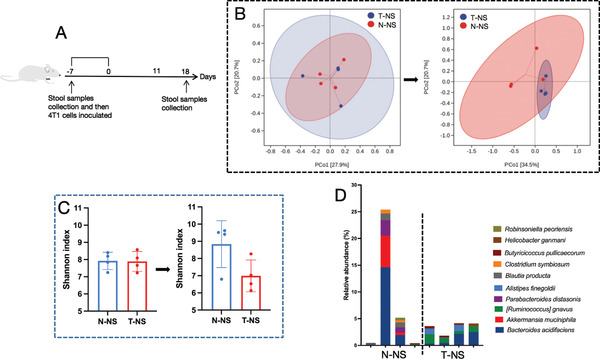
Differences in the microbial community. A) Outline of the mouse experiment (*n* = 4). B) PCA data showing that the intestinal microbiota changed with tumor growth. The picture on the left was taken before inoculation, and the picture on the right was taken after inoculation. C) Comparison of microbial diversity (Shannon index, mean ± SD, Student's *t*‐test). D) Composition and abundance of the intestinal microbiota at the species level.

According to the above intestinal studies, we hypothesized that the diversity and composition of the intestinal microbiota may affect tumor growth. To test this hypothesis, we compared the intestinal microbiota between tumor‐bearing and normal mice. The collection time of the stool samples is shown in **Figure** **9**A. We observed that the microbial community changed with tumor development (Figure 9B). Moreover, the microbial diversity (Shannon index) of normal mice increased, while that of tumor‐bearing mice decreased (Figure 9C). Previous data have also shown that the intestinal microbiota diversity of autoimmune hepatitis patients was characterized by lower diversity than those of healthy controls.^[^ ^56^ ^]^ Therefore, we suspect that disease alters the diversity of the intestinal microbiota. In addition, the abundance of *A. muciniphila* was elevated in the normal mice, while that of *R. gnavus* was elevated in the tumor‐bearing mice (Figure 9D).

### 2.5 Tumor Growth Inhibition In Vivo

**Figure 10:**
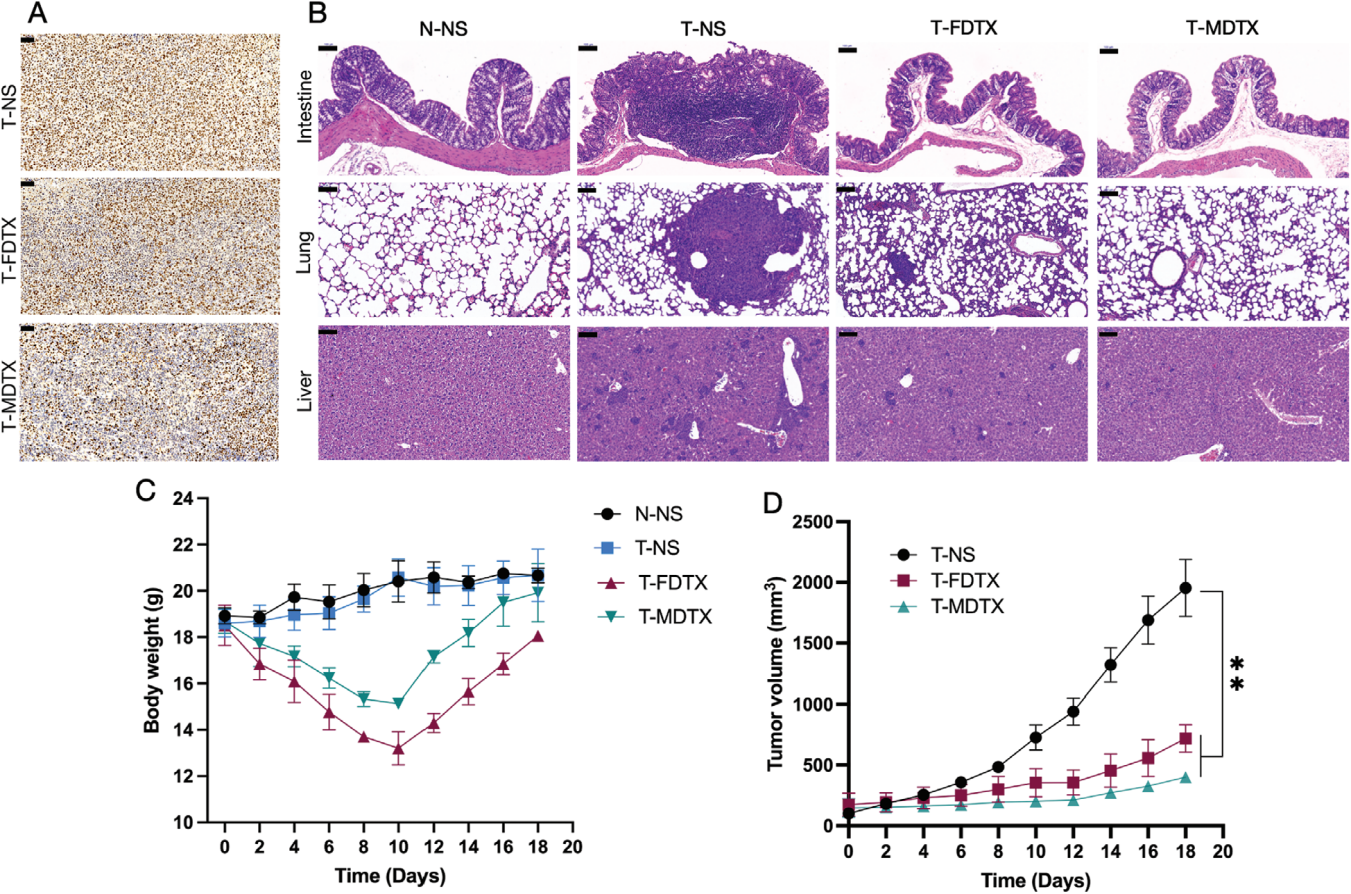
Antitumor efficacy of DTX micelles and free DTX. A) Tumor sections stained for Ki‐67 are shown (scale bar, 50 µm). B) H&E staining showing tissue damage (scale bar, 100 µm). C) Changes in body weight in different groups of mice (*n* = 4, mean ± SD, Student's *t*‐test). D) Tumor sizes of the mice in each group (*n* = 4, mean ± SD, Student's *t*‐test).

Immunohistochemical staining of tumor sections for the Ki‐67 antigen revealed that DTX micelle‐treated mice exhibited lower Ki‐67 expression than free DTX‐treated mice (**Figure** **10**A). Hematoxylin and eosin staining (H&E) staining was used to evaluate colon, lung, and liver damage in normal saline‐, free DTX‐ and DTX micelle‐treated mice, and DTX micelle‐treated mice exhibited less damage than the mice in the first two groups (Figure 10B).

We recorded the body weight and tumor size of the mice after administration. After the second injection of free DTX or DTX micelles, the mice began to lose weight, and the free DTX‐treated mice showed the most obvious weight loss (Figure 10C). The body weight was the lowest on the 12th day after the first injection and then gradually recovered. The tumor volume of the DTX micelle‐treated mice was smaller than that of the free DTX‐treated mice (Figure 10D). These results showed that the nanoparticles have advantages in improving efficacy and reducing toxicity.^[^ ^57^ ^]^

## 3 Conclusion

In summary, the present study demonstrates that the DTX nanoparticle drug‐loading system reduces intestinal barrier damage and is more beneficial for intestinal microbiota diversity enhancement and probiotic enrichment than free DTX. In addition, upon comparing the intestinal microbiota of antibiotic mixture‐treated mice and normal saline‐treated mice, tumor‐bearing mice, and normal mice, we found that an intestinal microbiota with high diversity is more likely to contribute to antitumor activity than one with low diversity. Although many of the details governing the complex interplay among the antitumor efficacy of DTX micelles, gastrointestinal side effects, intestinal barrier integrity, intestinal microbial diversity, and composition remain to be deciphered, the present study reveals that the DTX micellar formulation is beneficial to intestinal barrier integrity, intestinal microbiota diversity, and probiotic enrichment. Overall,these results suggest that non‐orally administrated DTX also affects the intestinal barrier and intestinal microbiota. This conclusion contributes to the body of knowledge regarding side effects to aid in the development of nanomedicines and may contribute to the development of safer and more effective nanomedicines for the treatment of cancer and other diseases.

## 4 Experimental Section

### Synthesis of DTX Micelles

DTX micelles were prepared according to the methods in previous reports from the authors’ laboratory.^[^ ^3^ ^]^ The diameter of the DTX micelles was determined with a Malvern Zetasizer Nano ZS (Malvern Nano ZS 90, Malvern, UK), and the morphology of the DTX micelles was detected by TEM.

### Cell Culture

The Caco‐2 cell line (human colorectal adenocarcinoma cell line) and the 4T1 cell line (murine breast cancer cell line) were purchased from the American Type Culture Collection (ATCC) and cultured with DMEM and RPMI 1640 medium, respectively. The cells were grown in a 37 °C incubator with a humidified 5% CO_2_ atmosphere.

### Animals

Female Balb/c mice at 5–6 weeks of age were purchased from HFK Bioscience Co., Ltd. (Beijing, China) and given with free access to standard food and water under specific pathogen‐free conditions. All animal procedures were approved by the Institutional Animal Care and Treatment Committee of Sichuan University (Chengdu, P. R. China).

### Fluorescence In Vivo

The distributions of free DTX and DTX micelles in vivo and in the intestine were investigated. 4T1 cells (1 × 10^6^ per mouse) were injected into the right flange of female Balb/c mice. The near‐infrared fluorescent dye DiD was used to replace DTX in the micelles and evaluate the micellar distribution in vivo. The injection dose of DiD in free DiD‐treated mice and DiD micelle‐treated mice was 100 µg kg^−1^. The fluorescence intensity of DiD in tumors was detected with a fluorescence imaging system (IVIS Lumina Series III, PerkinElmer, USA; excitation = 645 nm, emission = 715 nm) at predetermined time points.^[^ ^58^ ^]^ After that, the mice were sacrificed, intestinal tissue was collected, and the fluorescence intensity was evaluated. In addition, the colon was collected and immediately frozen at −80 °C until it was sliced. After slicing, and the distribution of DiD in the intestine was observed by confocal microscopy (ZEISS LSM 880).

### In Vivo Barrier Assessment

Based on the accumulation of DTX in the intestine, the impact on the intestinal barrier was analyzed. Mice were injected subcutaneously with 4T1 cells (1 × 10^6^ per mouse) for the tumor growth experiment. When the tumor volume reached ≈100 mm^3^, the mice were randomly divided into three groups (*n* = 4) and then intravenously injected with normal saline, free DTX (dose of DTX: 10 mg kg^−1^). and DTX micelles (dose of DTX: 10 mg kg^−1^), respectively. The mice were injected every other day three consecutive times. Intestinal permeability was measured on the 7th day after administration. After fasting for 4 h, the mice were fed FITC‐dextran (440 mg kg^−1^ body weight), and blood was collected 4 h later. Serum was separated from the blood, and the concentration of FITC‐dextran in the serum was determined with a fluorimeter with an excitation wavelength of 485 nm and an emission wavelength of 528 nm. In addition, colons were collected and immediately frozen at −80 °C until RNA was extracted, and the expression of ZO‐1 and occludin was examined. Additional colon tissues were collected and fixed with 4 wt% paraformaldehyde for 2 days, ZO‐1 and occludin staining was performed as described previously.^[^ ^3^ ^]^

### In Vitro Barrier Assessment

The Caco‐2 monolayer is the most common in vitro model of the intestinal epithelium.^[^ ^59^, ^60^ ^]^ Caco‐2 cells were seeded in 6‐well plates (1 × 10^5^) and allowed to grow for 72 h to form Caco‐2 monolayers. The cells were treated with normal saline, free DTX, or DTX micelles for 24 h, and the morphology of the Caco‐2 cells was observed by light microscopy (Bio‐Rad). Then, the cells were collected and stored at −80 °C.

Caco‐2 cells were grown on glass cover slips to form Caco‐2 monolayers and treated with normal saline, free DTX, or DTX micelles for 24 h. The cells were fixed with 4% wt polyformaldehyde. Tight junction labeling was performed with anti‐ZO‐1 and anti‐occludin antibodies (Bioss). The cells were imaged using a fluorescence microscope (ZEISS).

### RNA Isolation and qPCR

The Caco‐2 cells and colon tissues were removed from −80 °C, and RNA was extracted using an E.Z.N.A Total RNA Kit I (Omega Biotek) according to the protocol. Reverse transcription was performed using a cDNA Reverse Transcription Kit (Takara). qPCR was performed with SYBR green qPCR Mix (TIANGEN) on a CFX96 (BiO‐RAD). The cycling conditions were 95 °C for 15 min, followed by 40 cycles of 95 °C for 10 s, 58 °C for 30 s, and 72 °C for 30 s. The primers used for amplification were described.^[^ ^42^ ^]^

### Differences in the Intestinal Microbiota

To compare the effects of free DTX and DTX micelles on the intestinal microbiota, mice were injected subcutaneously with 4T1 cells and randomly divided into three groups (*n* = 4), mice were intravenously injected with normal saline, free DTX (dose of DTX: 10 mg kg^−1^), and DTX micelles (dose of DTX: 10 mg kg^−1^). Each mouse was injected every other day three consecutive times, and stool samples were collected 7 days after administration.

In addition, to verify the effect of the intestinal microbiota on tumor growth in mice, mice were randomly divided into two groups (*n* = 4). In the first group, the mice were administered 100 µL of an antibiotic mixture (ampicillin, 1 mg mL^−1^; gentamicin, 1 mg mL^−1^; metronidazole, 1 mg mL^−1^; neomycin, 1 mg mL^−1^; and vancomycin, 0.5 mg mL^−1^); in the second group, the mice were administered 100 µL of normal saline. Three consecutive doses were given before 4T1 cell inoculation. Stool samples were collected 5 days after 4T1 cell inoculation. Stool samples from normal and tumor‐bearing mice (*n* = 4) before and 25 days after inoculation with 4T1 cells were also collected and compared the changes in the intestinal microbiota during tumor development. All stool samples were immediately frozen at −80 °C until the DNA was extracted.

DNA was extracted with an isolation kit (OMEGA Stool DNA Kit (50), D4015‐01) following the protocol described for the kit. DNA was assessed with a NanoDrop 2000 (Thermo, USA) to determine the concentration and purity.^[^ ^61^ ^]^ The V3–V4 region of the 16S rRNA gene was amplified using 338F and 806R as primers (338F, 5′‐ACTCCTACGGGAGGCAGCA‐3′; 806R, 5′‐GGACTACHVGGGTWTCTAAT‐3′),^[^ ^62^ ^]^ and Illumina MiSeq sequencing was used to perform microbial composition analysis.

### 16S rRNA Gene Sequence Analysis

OTUs were defined using a 97% similarity cutoff for all data and were clustered with QIIME2.^[^ ^63^ ^]^ QIIME (version QIIME2) was also used to calculate the Shannon index, to perform PCA, and to calculate the relative abundance values of intestinal microbes at the genus and species levels. The Shannon index was used to evaluate the diversity of the intestinal microbiota. PCA was used to analyze the similarities and differences of the intestinal microbiota in different groups. The KEGG pathway/module profile was analyzed with PICRUSt (version PICRUSt2), and 16S rRNA marker genes were used to predict the microbial community function.^[^ ^64^ ^]^

### Antitumor Effect In Vivo

The antitumor activity of the drugs was investigated in a subcutaneous 4T1 model. There were four mice in each group. The tumor volumes and body weights were measured every other day and calculated with the formula: volume (mm^3^) = length × width^2^ × 0.5. The proliferation of tumor cells was analyzed by immunohistochemical staining of Ki‐67 (LabVision, MA, USA), which was performed as described previously.^[^ ^3^, ^65^ ^]^ To evaluate the development and severity of the tumors, organs were collected and fixed in 4% wt paraformaldehyde for 2 days, and H&E staining was used to visualize organ damage.^[^ ^66^, ^67^ ^]^ Images were captured with a Pannoramic MIDI.

### Statistical Analysis

Statistical analyses were performed using the GraphPad Prism 8 (GraphPad Software Inc.). This software was used to perform Student's *t*‐test. All results were presented as the mean value ± standard deviation (SD). There was statistical significance at *p* < 0.05 (^*^ *p* < 0.05, ^**^ *p* < 0.01, ^***^ *p* < 0.001).

## Conflict of Interest

The authors declare no conflict of interest.

## Author Contributions

Q.Y.L., J.R.P., and Z.Y.Q. designed experiments; Q.Y.L., L.Y., and Y.X. performed experiments; Z.Y.Q., J.R.P., D.R.H., and Y.H., provided technical support; Q.Y.L., R.X.H., and L.P.Y., analyzed data; Q.Y.L., J.R.P., and Z.Y.Q., wrote and revised the manuscript. All authors have approved the final manuscript.

## Data Availability

The 16S rRNA gene sequences have been deposited in the NCBI Sequence Read Archive (SRA) database with the accession numbers SRP237470 and SRP278881.
